# A new species of the genus *Rana* from Henan, central China (Anura, Ranidae)

**DOI:** 10.3897/zookeys.694.12513

**Published:** 2017-08-29

**Authors:** Haipeng Zhao, Junxiao Yang, Chunping Wang, Pipeng Li, Robert W. Murphy, Jing Che, Zhiyong Yuan

**Affiliations:** 1 Kunming Institute of Zoology, Chinese Academy of Sciences, Kunming 650223, Yunnan, China; 2 Kunming College of Life Science, University of Chinese Academy of Sciences, Kunming 650204, Yunnan, China; 3 School of Life Science, Henan University, Kaifeng 475004, Henan, China; 4 Henan Forestry Survey and Planning Institute, Zhengzhou 450045, Henan, China; 5 Center for Chinese Endemic Herp-breeding and Conservation Research, and Liaoning Key Laboratory of Evolution and Biodiversity, Shenyang Normal University, Shenyang 110034, Liaoning, China; 6 Centre for Biodiversity and Conservation Biology, Royal Ontario Museum, 100 Queen’s Park, Toronto M5S 2C6, Canada; 7 College of Forestry, Southwest Forestry University, Kunming 650224, Yunnan, China

**Keywords:** Brown frog, DNA barcode, genealogy, *Rana luanchuanensis* sp. n.

## Abstract

A new species of brown frog *Rana luanchuanensis* Zhao & Yuan, **sp. n.** is described from Luanchuan County, western Henan, central China. The mitochondrial genealogy suggests that the new species is the sister taxon to the clade including *R. amurensis* and *R. coreana*, and is separated by uncorrected pairwise distances more than 12.5%. Morphologically, this new species differs from its congeners by a suite of characters. Analyses of partial sequences of cytochrome oxidase subunit I (COI) resolve the new species as a single matriline.

## Introduction

Frogs in the genus *Rana* Linnaeus, 1758 (type species: *Rana temporaria* Linnaeus, 1758), are commonly known as brown or wood frogs. Currently, the genus *sensu* Yuan et al. (2016) contains 101 species (AmphibiaWeb 2017). It is a widespread, complex, and diverse group that crosses Eurasia and the Americas. They share prominent dorsolateral folds, a dark temporal mask, and a body that is counter-shaded in various shades of brown, which lead to the common English name “brown frogs” (Boulenger 1920; Liu and Hu 1961). The conservative morphology of Eurasian *Rana* makes many species notoriously difficult to identify (Che et al. 2007a, b; Liu and Hu 1961). In this case, molecular assessments have resulted in the description of new species (e.g. Lu et al. 2007; Yan et al. 2011; Matsui 2011; Ryuzaki et al. 2014). Additional cryptic new species were suggested to occur in the New World (Hillis and Wilcox 2005).

**Figure 1.**
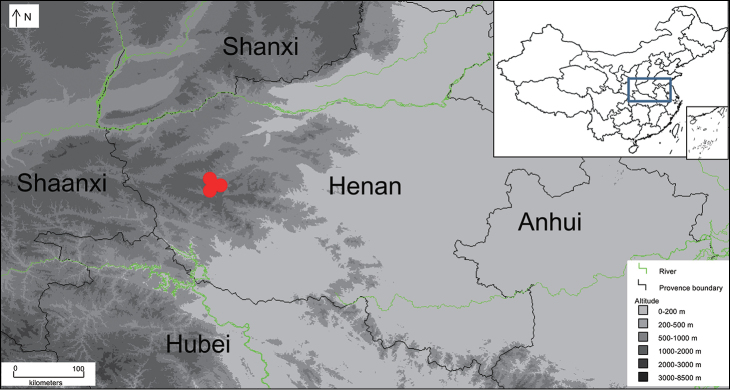
Map showing the collecting locations of *Rana luanchuanensis* sp. n. indicated by red cycles.

Five of seven clades of *Rana* (Yuan et al. 2016) exist in China (AmphibiaChina 2017), including 23 species. Among others, *R. chensinensis* David, 1875, *R. culaiensis* Li, Lu & Li, 2008, and *R. zhenhaiensis* Ye, Fei, & Matsui, 1995 occur in Henan (AmphibiaChina 2017). Recent herpetofaunal surveys in Henan (August 2007, November 2013, and May 2014) led to the discovery of three new populations of *Rana* in western areas (Figure 1). These populations show distinct and curved dorsolateral folds and their males do not possess subgular vocal sacs. These characters are similar to those of the *R. amurensis* species group, which contains *R. amurensis* Boulenger, 1886 and *R. coreana* Okada, 1928 (Fei et al. 2009; Yang et al. 2010; Zhou et al. 2015). Further, these frogs possess several distinct morphological characters that differ from *R. amurensis* and *R. coreana*. Taken together, these data suggest that the new populations might be a new species.

Herein, the identity of a new brown frog is investigated by comparing morphological and molecular characteristics with Eurasian congeners. Analyses determine that the frogs constitute a new species, which is described here.

## Materials and methods

### Sampling

From 2013 to 2014, field surveys conducted in Luanchuan, western Henan resulted in the collection of 38 adult frogs. Following euthanizing, muscle or liver tissue was dissected from specimens and then preserved in 95% ethanol. Voucher specimens were fixed in 10% buffered formalin, and then later transferred to 70% ethanol. All specimens were deposited in the Kunming Institute of Zoology (KIZ), Chinese Academy of Sciences. Tissues samples used in our comparative analyses were summarized in Table 1, along with locality data, voucher numbers, and GenBank accession numbers.

**Table 1.** Voucher specimens, localities, and GenBank accession numbers for brown frogs, *Rana*. “*” = type locality. GBN = GenBank Accession No.

Species	Voucher No.	Locality	GBN (COI)	Source
*Rana amurensis*	KIZ070423558	Shangzhi, Heilongjiang, China	JF939079	Yan et al. (2011)
*Rana asiatica*	XJ416	Forty seven Tuan, Xinjiang, China	MF149925	This study
*Rana arvalis*	KIZ04239	Haba River, Xinjiang, China	MF149926	This study
*Rana arvalis*	GBOL03518	Upper Bavaria, Bavaria, Germany	KP697924	Hawlitschek et al. (2015)
*Rana chaochiaoensis*	KIZ06425	Zhaojue, Sichuan, China*	JF939103	Yan et al. (2011)
*Rana chensinensis*	KIZRD05SHX01	Huxian, Shanxi, China*	JF939080	Yan et al. (2011)
*Rana coreana*	MMS223	South Korea	MF149928	This study
*Rana coreana*	KIZYPX2630	Mt. Kunyu, Shandong, China*	MF149927	This study
*Rana culaiensis*	KIZSD080501	Mt. Culai, Shandong, China*	JF939082	Yan et al. (2011)
*Rana dybowskii*	KlZ070423448	Huangnihe, Jilin, China	JF939078	Yan et al. (2011)
*Rana hanluica*	KIZYPXll72	Mt. Yangming, Hunan, China*	JF939099	Yan et al. (2011)
*Rana huanrensis*	SYNU040006	Huanren, Liaoning, China*	JF939072	Yan et al. (2011)
*Rana japonica*	KIZYPX11775	Japan	JF939101	Yan et al. (2011)
*Rana jiemuxiensis*	KIZ05263	Jiemuxi NR, Hunan, China*	JF939090	Yan et al. (2011)
*Rana kukunoris*	CJ06102001	Qinghai Lake, Qinghai, China*	JF939073	Yan et al. (2011)
*Rana longicrus*	KIZ15026	Nanzhuang, Miaoli, Taiwan, China	JF969067	Yan et al. (2011)
*Rana luanchuanensis* sp. n.	KIZ047393	Luanchuan, Henan, China*	MF149924	This study
*Rana luanchuanensis* sp. n.	KIZ047452	Luanchuan, Henan, China*	MF149923	This study
*Rana luanchuanensis* sp. n.	KIZ047476	Luanchuan, Henan, China*	MF149921	This study
*Rana luanchuanensis* sp. n.	KIZ047482	Luanchuan, Henan, China*	MF149920	This study
*Rana luanchuanensis* sp. n.	KIZ047487	Luanchuan, Henan, China*	MF149922	This study
*Rana luteiventri*	MVZ Herp 137417	Missoula, Montana, USA	KU985757	Chambers and Hebert (2016)
*Rana omeimontis*	KIZ02424	Mt. Emei, Sichuan, China*	JF939069	Yan et al. (2011)
*Rana zhenhaiensis*	KIZ0803271	Zhenhai, Zhejiang, China*	JF939065	Yan et al. (2011)
*Rana zhengi*	SCUM0405190CJ	Zhangcun, Hongya, Sichuan, China*	MF149929	This study

### DNA extraction, amplification, and sequencing

Total genomic DNA was extracted from tissues of five individuals using the standard phenol-chloroform protocols (Sambrook et al. 1989). Partial sequences of the gene encoding cytochrome oxidase subunit I (COI) was amplified and sequenced.

Amplification was performed in a 25 μL volume as follows: initial denaturation step for 5 min at 95 °C followed by 35 cycles of denaturation for 1 min at 94 °C, primer-specific annealing temperature of 46 °C for 1 min, extension for 1 min at 72 °C; final extension at 72 °C was conducted for 10 min. The primers Chmf4 (5’-TYTCWACWAAYCAYAAAGAYATCGG-3’) and Chmr4 (5’-ACYTCRGGRTGRCCRAARAATCA-3’) (Che et al. 2012) were used for amplification and sequencing. PCR products were purified with Gel Extraction Mini Kit (Tiangen Biotech, Beijing). The cycle sequencing reactions were performed using BigDye Terminator Cycle Sequencing Kit (v.2.0, Applied Biosystems, Foster City, California, USA), using purified products as the template DNA. Sequences were determined using an ABI PRISM 3730 automated DNA sequencer with sequencing in both directions. The sequence data were submitted to a BLAST search in GenBank to confirm the identity. Considering geography and morphological similarity, 13 COI sequences of Eurasian *Rana* were retrieved from GenBank and included in the subsequent molecular analyses (Table 1). *Rana luteiventris* was chosen as outgroup based on the phylogeny of Yuan et al. (2016). Nucleotide sequences were aligned using MUSCLE v.3.6 (Edgar 2004) with default parameters.

### Genetic analyses

Interspecific and intraspecific mean uncorrected pairwise distances were computed in MEGA v.6.0 (Tamura et al. 2013). Phylogenetic analyses of the sequences were conducted using Bayesian inference (BI) and maximum likelihood (ML). The BI analysis was executed in MrBayes v.3.1.2 (Ronquist and Huelsenbeck 2003) using GTR + I + G model determined using the Akaike Information Criterion (AIC) computed with jModelTest 2 (Darriba et al. 2012). Consensus frequencies, termed Bayesian posterior probabilities, were used to estimate nodal support. Four separate runs were performed with four Markov chains. Each run was conducted for 10000000 generations while sampling every 1000 generations. Log likelihood scores were tracked for stabilization and the first 50% of the trees were discarded as burn-in. The sampled trees were analyzed using Tracer v.1.6 (Rambaut et al. 2014) to confirm satisfactory convergence of topological split frequencies. The ML analysis was conducted using RAxML v.8.0 (Stamatakis 2014). This analysis implemented the GTR + I + G model. Nodal support values were estimated from 1000 nonparametric bootstrap pseudoreplicates.

### Morphometrics

A total of 38 specimens was examined (Appendix 1). Haipeng Zhao took all measurements of the specimens to minimize bias. Nineteen linear measurements following Fei et al. (2009) were made using digital dial calipers with a precision of 0.1 mm. These measurements were as follows:

**SVL** (snout-vent length),

**HDL** head length,

**HDW** head width,

**SL** snout length,

**EYE** diameter of exposed portion of eyeball,

**IOD** interorbital distance,

**IND** internarial distance,

**UEW** upper eyelid width,

**TYE** tympanum outer diameter,

**LAL** lower-arm length,

**HL** hand length,

**LAHL** lower-arm and hand length,

**LAW** lower-arm width,

**FEL** femur length,

**TL** tibia length,

**TW** tibia width,

**FTL** length of foot and tarsus,

**FOL** foot length, and

**IMTL** inner metatarsal tubercles length.

Dissections on five male specimens determined the presence or absence of vocal sacs which can be seen by the presence of openings on the mouth floor. Vocal sacs that are externally visible are defined as “external” vocal sacs, those that cannot be distinguished by external observation are defined as “subgular”. All these morphological characters are defined following Fei et al. (2009).

## Results

Ten new sequences with 558 base pairs (bp) were obtained and deposited in GenBank (Accession numbers MF149920–MF149929; Table 1). After trimming ends, the combined sequences contained 211 variable sites of which 187 were potentially parsimony-informative. The uncorrected *p*-distances between the new populations from Henan and congeners ranged from 12.54% (*R. amurensis*) to 17.92% (*R. longicrus*) (Table 2). The uncorrected pairwise distances between the two new populations from Henan were less than 0.1%.

**Table 2.** The pairwise uncorrected *p*-distance (%) of the COI partial sequence used in this study (a, b). 1: *Rana amurensis*; 2: *R. asiatica*; 3. *R. arvalis*; 4: *R. chaochiaoensis*; 5: *R. chensinensis*; 6: *R. coreana*; 7: *R. culaiensis*; 8: *R. dybowskii*; 9: *R. hanluica*; 10: *R. huanrensis*; 11: *R. japonica*; 12: *R. jiemuxiensis*; 13: *R. kukunoris*; 14: *R. longicrus*; 15: *R. luanchuanensis* sp. n.; 16: *R. omeimontis*; 17: *R. zhenhaiensis*; and 18: *R. zhengi*. Bolded number highlights the distance between *R. luanchuanensis* sp. n. and the species of *Rana* analyzed in this study. “—” indicates genetic distance less than 1%.

**(a)**									
	**1**	**2**	**3**	**4**	**5**	**6**	**7**	**8**	**9**
1	—								
2	0.1562	—							
3	0.1595	0.1113	—						
4	0.1631	0.14	0.1326	—					
5	0.1523	0.1436	0.1452	0.1613	—				
6	0.1093	0.1609	0.1455	0.1553	0.1699	0.0164			
7	0.1828	0.1364	0.1685	0.1308	0.1685	0.1843	—		
8	0.1685	0.1311	0.138	0.1595	0.1326	0.1626	0.1774	—	
9	0.1756	0.14	0.1577	0.1272	0.1595	0.1824	0.0717	0.1685	—
10	0.1487	0.1329	0.1344	0.1541	0.0502	0.1726	0.1703	0.129	0.1649
11	0.1685	0.1382	0.147	0.1308	0.1416	0.1518	0.147	0.1541	0.1308
12	0.172	0.14	0.1613	0.1398	0.1559	0.1798	0.0771	0.1756	0.0771
13	0.1613	0.1346	0.1487	0.1541	0.0609	0.1698	0.1703	0.1308	0.1649
14	0.1792	0.1472	0.1738	0.1434	0.1756	0.1852	0.0287	0.1792	0.0878
**15**	**0.1254**	**0.1346**	**0.1523**	**0.1523**	**0.1452**	**0.1464**	**0.1649**	**0.1505**	**0.1703**
16	0.1631	0.1436	0.1487	0.1487	0.1613	0.1725	0.0806	0.1505	0.0771
17	0.1846	0.1508	0.1685	0.138	0.1667	0.1969	0.0323	0.181	0.0806
18	0.1667	0.1382	0.1416	0.1452	0.1434	0.1716	0.1703	0.1523	0.1559
**(b)**									
	**10**	**11**	**12**	**13**	**14**	**15**	**16**	**17**	**18**
11	0.1416	—							
12	0.1613	0.1487	—						
13	0.0484	0.1452	0.1577	—					
14	0.181	0.1541	0.086	0.181	—				
**15**	**0.1434**	**0.1362**	**0.1792**	**0.1487**	**0.1738**	—			
16	0.1649	0.1559	0.0932	0.1559	0.0932	**0.1559**	—		
17	0.181	0.1416	0.086	0.181	0.043	**0.1667**	0.0806	—	
18	0.1577	0.1667	0.1667	0.1541	0.1774	**0.1685**	0.1649	0.1703	—

**Figure 2.**
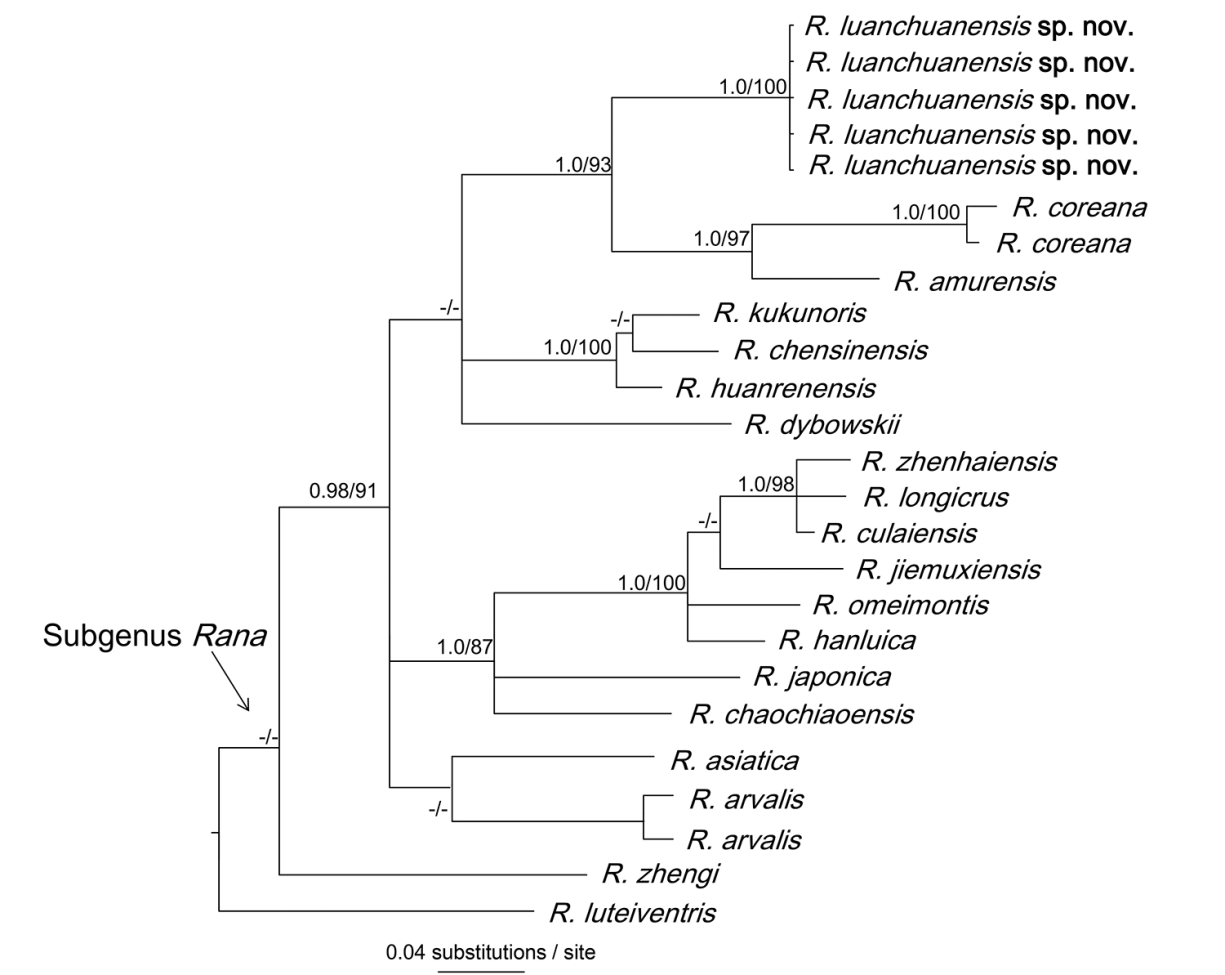
A Bayesian inference tree based on the COI partial sequence data. Numbers near the nodes are Bayesian posterior probabilities / ML bootstrap value but only when values are ≥ 0.95 and ≥ 70, respectively.

Genealogical reconstructions by BI and ML were nearly identical (Figure 2). The monophyly of ingroup and major clades were similar to previous studies (Yan et al. 2011; Yuan et al. 2016). However, phylogenetic relationships among the five major clades were not recovered by our analyses likely due to limited data; future study using additional loci were deemed to be desirable. The new samples from Luanchuan, Henan shared a common matriline, which clustered as the sister-group of *R. amurensis* plus *R. coreana* with strong support (BI BPP= 100, ML BS = 93). This resolution and the extent of sequence divergence suggested that the new samples constituted a new species.

Morphological and morphometric analyses of the frogs (Table 3) identified several diagnostic morphological characters (see below).

**Table 3.** Linear measurements (in mm) of *Rana luanchuanensis*. The abbreviations are provided in text.

Character	Measurements		
		♂	♀
SVL	Range	27.2–33.0 ± 1.87	23.7–41.2 ± 4.25
HDL	Range	7.9–10.5 ± 0.69	8.5–13.5 ± 1.25
HDW	Range	9.7–11.8 ± 0.63	9.7–15.2 ± 1.47
SL	Range	4.3–5.7 ± 0.34	4.1–6.9 ± 0.7
EYE	Range	2.9–4.3 ± 0.35	3.3–5.3 ± 0.55
IOD	Range	2.4–3.4 ± 0.3	2.5–4.3 ± 0.39
IND	Range	2.3–3.6 ± 0.29	2.5–3.4 ± 0.27
UEW	Range	1.9–2.6 ± 0.26	1.9–3.1 ± 0.36
TYE	Range	1.4–2.4 ± 0.25	1.4–3.2 ± 0.44
LAL	Range	5.9–7.4 ± 0.44	5.3–8.5 ± 0.78
HL	Range	7.2–9.1 ± 0.54	6.8–11.2 ± 1.11
LAHL	Range	13.3–15.3 ± 0.6	11.7–18.2 ± 1.7
LAW	Range	2.3–3.6 ± 0.4	1.7–3.8 ± 0.5
FEL	Range	13.9–18.2 ± 1.35	13.5–21.7 ± 2.1
TL	Range	15.5–20.3 ± 1.35	14.7–22.8 ± 2.18
TW	Range	2.5–4.1 ± 0.52	2.1–4.9 ± 0.7
FTL	Range	21.0–26.4 ± 1.36	19.7–33.3 ± 3.26
FOL	Range	15.8–18.6 ± 0.93	13.2–23.4 ± 2.52
IMTL	Range	1.7–2.4 ± 0.23	1.4–2.8 ± 0.28

### Rana luanchuanensis

Taxon classificationAnimaliaAnuraRanidae

Zhao & Yuan sp. n.

http://zoobank.org/3FF49AB8-95A4-4C30-8735-2B0C5778A683

Figures 3 , 4

#### Holotype.

KIZ016090, an adult male, collected by Haipeng Zhao and Ruiliang Wang on 4 May 2014 in Tongyi River near the village of Hanqiu (33.80°N, 111.80°E, elevation 810 m a.s.l.), Miaozi town, Luanchuan County, western Henan, central China.

#### Paratypes.

KIZ047446–KIZ047453, KIZ016089, with the same collection data as the holotype; KIZ047383, with the same locality as the holotype, collected on 16 November 2013; KIZ047470–KIZ047487, KIZ016086–KIZ016088, and KIZ016091 from Wangping village, Tantou town, Luanchuan County (33.95°N, 111.73°E, elevation 530 m a.s.l.) in the same river, collected on 5 May 2014; KIZ0093, KIZ0099, KIZ0101, KIZ0104, and KIZ0105, collected by Li Ding and Xiaobei Zhang from nearby Miaozi town (33.75°N, 111.72°E, elevation 1070 m a.s.l.) on 15 August 2007. A total number of 37 adult individuals included 12 males and 25 females.

#### Diagnosis.

A small-sized species (SVL 27.2–33.0 mm in males; 23.7–41.2 mm in females) of *Rana*; temporal fold distinct; dark mask covering tympanum; curved dorsolateral fold thin, extending from posterior canthus to groin; tips of fingers not expanded; skin smooth with few small granules on dorsum and legs, distinct large tubercles absent; head length slightly less than head width; vocal sac absent in males; white rictal gland absent on the upper lip; ventral surface of throat, chest, and belly white with irregular black spots; poster part of abdomen and ventral surface of thighs and limbs reddish; distinct transverse grayish brown bars on dorsal surface of fingers and toes, lower arms, tarsus, thighs, and tibia; toes two-thirds webbed; gray-blackish nuptial pad prominent and forming two groups in males, with minute nuptial spines; three metacarpal tubercles, inner one close to the nuptial pad at the base of finger I, the two outer ones closed together at the base of fingers III and IV.

#### Description of holotype.

SVL 32.8 mm. *Head* slightly shorter than broad (HL\HW = 0.87), snout pointed and projecting; snout length much longer than eye diameter (SL\EYL = 1.35); interorbital space equal to internasal space and both wider than upper eyelid width; tympanum diameter about half of eye diameter, loreal region concave, sloping outwards; vomerine teeth in short oblique series, anterior edges in line with centers of choanae; tongue deeply notched posteriorly; vocal sacs absent.

*Forearm* robust, fingers slender, unwebbed; tips of fingers not expanded, with no circum-marginal grooves; relative length of fingers: II < I < IV < III; one prominent subarticular tubercle on fingers I and II, two small subarticular tubercles on fingers III and IV; distinct supernumerary tubercles below the base of fingers; inner metacarpal tubercle strong and large, ovoid, close to the nuptial pad at base of finger I; two outer tubercles close together at base of fingers III and IV, flat, long elliptic and obvious. Nuptial pad covered densely by small grey-blackish spines and divided into two groups, one near tip larger than the other one.

*Hindlimb* long (8.7 mm), heels well overlapping when limb held at right angles to body; tibiotarsal articulation of adpressed limb reaching far anterior to eyes; inner metatarsal tubercle weak and small, smooth, about 0.37 of the first toe; tips of toes similar to fingers; relative length of toes: I < II < III < V < IV; toes two-thirds webbed, webbing formula: I 1–2 II 1–2 ½ III 2–3 IV 3–1 V; web of toe III reaching the first joint from tip and on other toes nearly extending to tip; subarticular tubercles small, but visible; distinct supernumerary tubercles below the base of toes; inner metatarsal tubercle ovoid, small but distinct; outer metatarsal tubercle absent.

*Skin* rather smooth, except for some small granules near vent and ventral femoral region; temporal fold distinct, extending from posterior margin of eye above and behind tympanum to above arm insertion, a large triangular black and brown patch behind the eye and anterior to temporal fold; thin dorsolateral fold from posterior canthus to groin, obviously curved at upper tympanum and crossing temporal fold; ventral surface smooth, reddish; few granules on the posterior ventral surface of thighs.

**Figure 3.**
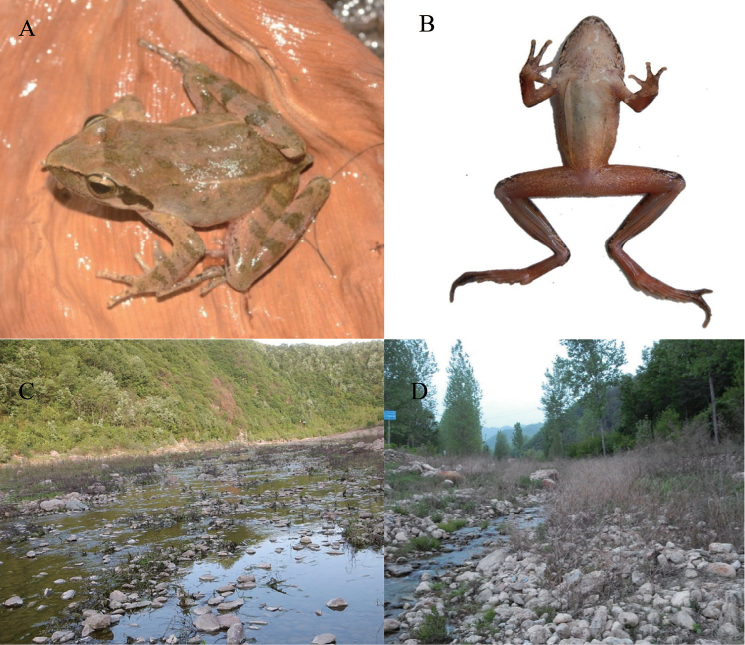
Photographs of a live specimen and its habitat near the type locality of *Rana luanchuanensis* sp. n. **A** Lateral view **B** Ventral view **C, D** Habitat of the type locality of *R. luanchuanensis* showing a live individual.

#### Color of holotype.

**Figure 4.**
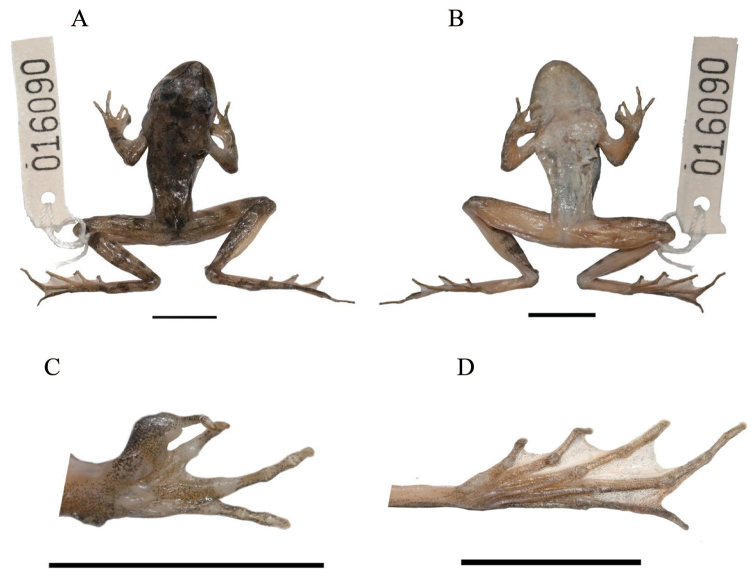
Holotype (KIZ016090) of *Rana luanchuanensis* sp. n. **A** Dorsal view **B** Ventral view **C** Details of left hand showing the nuptial pad **D** Details of left foot showing the extent of webbing. Scale bar 10 mm.

In life, dorsum gray-brown, with few scattered black spots and grayish brown blotches; dorsolateral fold reddish brown and darker than ground dorsal color; distinct grayish brown crossbars on dorsal surface of fingers and toes, lower arms, tarsus, thighs and tibia; narrow black stripe on edge of canthus rostralis from tip of snout along margin of upper eyelid and across eye continuing along supratympanic ridge; large triangular black and brown patch behind the eye and anterior to temporal fold; lower lip whitish with black spots and bars; throat, chest, and belly white with irregular black spots; poster part of abdomen reddish; ventrally limbs reddish with faint yellow nebulous mottling; faint yellow granules on ventral thigh; foot webbing brownish red with few indistinct black spots; nuptial pad blackish gray. In preservative, dorsal surface dark gray-brown with slightly paler limbs (Fig. 4); all grayish brown crossbars and grayish brown blotches fade to black; throat, chest, and abdomens fade to creamy white, with gray spots.

#### Habitat and life history.

All specimens of the new species were collected in shallow slow-flowing streams with large gravel beds (Figure 3). Streams were near the mountains with well-preserved forests. Females with mature eggs were collected from Hanqiu village on 16 November 2013, which indicated its breeding season may occur in the winter. However, we did not observe any breeding pairs, egg clutches, or tadpoles. More field work is needed to observe its breeding behavior and other phenomena of life history.

#### Variation in the type series.

Morphometric data were summarized in Table 3. Individuals varied in their dorsal ground color by ranging from being pinkish orange to dark brown. Number and shape of the spots and grayish brown blotches on dorsum varied. Number of grayish brown crossbars on dorsal surface of fingers, toes, lower arms, tarsus, thighs, and tibia varied. Forearm are much more robust in males than in females; nuptial pads are absent in females.

#### Etymology.

The specific epithet “luanchuanensis” is in reference to the type locality.

#### Comparisons.

*Rana luanchuanensis* sp. n. closely resembles the *R. amurensis* Boulenger, 1886 and *R. coreana* Okada, 1928, within the *R. amurensis* species group, but differs from them by the following morphological characters: 1) skin smooth (vs. many tubercles on the dorsum and dorsolateral surfaces of *R. amurensis* and many tubercles on the dorsolateral surface of *R. coreana*); 2) upper white rictal gland absent (vs. present in *R. amurensis* and *R. coreana*); 3) small size, SVL 27.2–33.0 mm in males and 23.7–41.2 mm in females (vs. SVL 48.8–66.4 mm in males and 51.2–70.4 mm in females of *R. amurensis*); 4) nuptial pad forming two groups in males (vs. nuptial pad forming four groups in males of *R. amurensis*); 5) toes two-thirds webbed (vs. toes half webbed in *R. coreana*); 6) transverse grayish brown bars on dorsal surface of fingers and toes, lower arms, tarsus, thighs, and tibia (vs. absent in *R. coreana*); and 7) ventral surface of throat, chest, and belly white with irregular black spots (vs. absence of black spots in *R. coreana*).

#### Distribution.

The species is currently only known from Luanchuan, Henan, China.

## Supplementary Material

XML Treatment for Rana luanchuanensis

## Appendix 1

**Specimens examined**

*Rana luanchuanensis* (n = 38): KIZ016090, KIZ04744-53, KIZ016089, KIZ047383, KIZ047470-87, KIZ016086-88, KIZ016091, KIZ023276, KIZ023282, KIZ023284, KIZ023287-88.
